# Personalized Nutrition in Food Allergy: Tips for Clinical Practice

**DOI:** 10.3389/fped.2020.00113

**Published:** 2020-03-27

**Authors:** Enza D'Auria, Erica Pendezza, Gian Vincenzo Zuccotti

**Affiliations:** Department of Pediatrics, Vittore Buzzi Children's Hospital, University of Milan, Milan, Italy

**Keywords:** food allergy, patient-tailored nutrition, oral food challenge, threshold dose, diet history

## Abstract

Nowadays, food allergies are considered as a wide spectrum of disorders that need different approaches. The “one size fits all” approach is giving way to a “targeted approach,” based on the identification of the patient's phenotype. Thus, the approach of nutritional management of food allergy has moved on from simply being “yes or no” to “how much?”, “in which form?” and “for which patients?” Different factors should be considered in order to make a patient-tailored nutritional plan in clinical practice. Tailored nutritional plans may help to reduce the nutritional, social and economic burden of food allergy.

## Introduction

While the mainstay treatment of food allergy remains allergen avoidance, food allergies are now considered as a wide spectrum of disorders that need different approaches (1–3).

**Figure 1:**
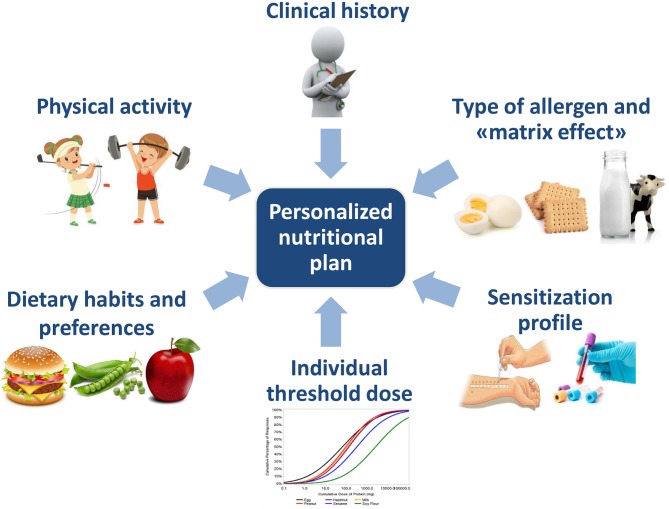
Factors to consider in order to make a personalized nutritional plan.

The “one size fits all” approach is giving way to a “targeted approach,” based on the identification of the patient's phenotype. Actually, different factors should be considered in order to make a patient-tailored nutritional plan in clinical practice (Figure 1). Other factors, like genetics and microbiota signature are likely be taken into account in the future (4).

Tailored nutritional plans may help to reduce the nutritional, social and economic burden of food allergy.

### Patient Sensitization Profile: Cross-Reactivity or Co-sensitization?

One of the main goals of the patient-tailored nutritional approach is to avoid unnecessary dietary restrictions.

The case of nuts is an example of how the approach to food allergy management has changed in recent years.

Until the last decade, the typical approach to children with tree nut or peanut allergy was exclusion from the diet of all types of nuts, in order to avoid risk of cross-reactivity or cross-contamination during processing (5). In 2014, the British Society for Allergy and Clinical Immunology (BSACI) recommended active inclusion in the diet of all types of tolerated nuts (6).

More recently, Couch et al. demonstrated that cross-reactivity among nuts, as proven by oral food challenge (OFC), may be lower than 30% (7). Thus, people who are allergic to one type of tree nut, such as cashews, may not be allergic to all other kinds of tree nuts.

From a nutritional standpoint, tree nuts *per se* represent a good source of energy, proteins, and minerals; thus, the reintroduction of certain types of nuts allows patients to include a wide range of foods and packaged products in their diet, which contributes to improving the patient's quality of life (8).

Fish allergy is another example of how hypersensitivity to one food within a species does not necessarily require the avoidance of the whole species.

Parvalbumin is the major fish allergen, responsible for more than 90% of IgE-mediated reactions and for more than 50% of clinical cross-reactivity among different fish species (9).

Two main isoforms have been identified: α-parvalbumin, which is not considered allergenic, and β-parvalbumin.

The clinical cross-reactivity between the two isoforms seems to be very low, both in cartilagineous and bony fish (10).

Parvalbumin content differs among various species of fish: in cartilagineous fish the α-isoform is predominantly expressed, while bony fish muscle mainly contains β-parvalbumin.

Also the concentration of parvalbumin can be very different among species: large migratory fish have a lower content than small sedentary ones.

For instance, tuna or swordfish parvalbumin content is <1 mg/g of fresh filet, whereas in cod and carp it is >2.5 mg/g (11).

This wide variability in parvalbumin content explains the different degrees of allergenicity: even children who are highly allergic to parvalbumin can tolerate one or more fish species with a lower content. Hence, fish allergy is not universal.

The implications for clinical practice are not to be neglected: consuming 2 portions of fish per week allows the child to introduce an adequate amount of EPA (eicosapentaenoic acid) and DHA (docosahexaenoic acid), without the need to use a dietary supplement (12).

Conversely, children who are not permitted to eat any type of fish should be supplemented with algal-derived omega-3 fatty acids (13).

The OFC remains the gold standard to confirm clinical reactivity, as it allows the allergist to test each single fish species and to provide personalized advices to the patient.

Also the approach to fruit and vegetable allergies can't be universal, since different sensitization profiles and clinical phenotypes exist.

Subjects with pollen-food syndrome (PFS) are primarily sensitized to pollen allergens (e.g., Bet v 1 homologs and profilins). In PFS, the ingestion of fresh fruits and vegetables often results in a mild clinical phenotype consisting of local and self-limiting reactions in the oropharynx, characterized by itching of the lips, mouth and throat with or without local angioedema, the so called oral allergic syndrome (OAS) (14).

In contrast, subjects with lipid-transfer protein (LTP) syndrome show a primary sensitization to plant food allergens (e.g., peach Pru p 3) (15).

Clinical expression of LTP sensitization can be variable: symptoms may range from mild local reactions (e.g., OAS) to severe systemic reactions, including anaphylaxis.

Symptoms of LTP hypersensitivity are often elicited when the assumption of the culprit food is concomitant with one or more cofactors, like fasting, exercise or drugs (e.g., non-steroidal anti-inflammatory drugs) (16).

In PFS, processed fruits, such as jam or cooked fruits, are well-tolerated and can be safely introduced into the child's diet as an important source of minerals, vitamins and dietary fiber.

LTPs are stable allergens that resist heat treatment and enzymatic digestion; thus, in case of LTP clinical relevant allergy, also processed forms of the culprit food should be strictly avoided (15).

Allergy to one or more LTP vegetable sources does not imply an allergy to other existing LTPs. Thus, only LTP-containing foods eliciting reactions must be excluded from the diet (16).

Component-resolved diagnostics can help the clinicians to distinguish between primary food allergy and OAS due to cross-reactivity to plant pollen and LTP clinical relevant allergy (17).

### Type of Allergen and the “Matrix Effect”: The Lesson From Milk and Egg

A large percentage of children who are allergic to cow's milk and hen's egg (70–75%) can tolerate the allergen if it is in an extensively heated form (18, 19).

Baked milk and egg are generally more tolerated not just because the antigen is underdosed (20).

Heat treatment reduces the allergenicity of many food proteins, probably by destructing the conformational epitopes of heat-labile proteins, increasing the effect of enzymatic digestion, and reducing intestinal absorption and basophil stimulation (21). However, the interactions between milk proteins and some components of the food matrix during heating seem to play the most important role in the reduction of allergenicity, thus limiting the accessibility of peptides to the immune system (22, 23).

“Baked” milk or egg refers to cookies, cakes, muffins, waffles, or other bakery products that contain cow's milk or hen's egg as an ingredient.

While it is well-accepted that some patients may tolerate baked egg and/or milk, there is no consensus yet about how to reintroduce baked products, meaning the setting in which to reintroduce them (e.g., at home or in hospital), and which food matrix is preferable (24).

In the last decade, allergen-component resolved diagnostics (CRD) has become increasingly used in the diagnostic work-up, aiding the clinicians to optimize decisions about if, when and how to perform OFC (25). In clinical practice, component resolved diagnostics (CRD) may be useful for evaluation of reactivity to baked milk, helping to identify different clinical phenotypes on the basis of molecular patient specific IgE profile. Patients who exhibit higher levels of casein-specific IgE are more likely to react to baked milk (26).

In turn, specific IgE diagnostic decision points for cow's milk allergy diagnosis (regular milk and heated milk, respectively) have been proposed (27–29). As well as the above, IgE for ovomucoid (Gal d 1) seems more predictive of reactivity to baked egg than sIgE to other epitopes (30). It is important to point out that positive and negative cut-offs are population specific; for this reason, they may not be relevant to all (31, 32).

In clinical practice, the use of decision points, combined with the patient clinical history, may allow the physician to identify optimal candidates to undergo OFC with baked milk. It is not possible at the moment to really predict which patients are able and which are unable to successfully pass challenges with baked milk and/or egg.

Generally, it is advisable to perform the oral provocation test under medical supervision, as the ingestion of cow's milk or hen's egg, even in baked form, can trigger symptoms, including severe and potentially life-threatening reactions (33).

Only in a few selected cases, characterized by undetectable or very low IgE levels toward heat stable epitopes, mild symptoms on accidental ingestion and no recent reaction, patients may be advised to gradually introduce baked milk at home (34).

A strict egg or milk-free diet causes important dietary restrictions, including the need for careful reading of labels (ingredients, trace allergens, hidden allergens) and the exclusion of numerous packaged foods (5).

Milk and egg are also contained in foods that are typically socially enjoyed by children, such as biscuits, cake and pizza; thus, the avoidance diet implies the difficulty of managing social events (20).

Indeed, liberalizing the diet- if possible- to baked products is associated with patients' and their families' improved quality of life, lower levels of stress and anxiety, and lower risk of an unhealthy approach to food (18).

### Individual Threshold Dose

Deriving patient threshold dose is necessary to personalize the nutritional advice. The true patient threshold lies between the No Observed Adverse Effect Level (NOAEL), the highest dose that will not produce any adverse effect in that person, and the Lowest Observed Adverse Effect Level (LOAEL), the lowest dose that produces an adverse effect (35). OFC actually remains the unique tool to determine individual threshold dose (36).

Although standardized protocols regarding the starting dose, the incremental doses and the intervals between doses exist (37, 38), it is important to remember that OFC is a diagnostic procedure and its design may be adapted, considering OFC indication and patient history (39).

Type of allergen, single or multiple allergy, age, sIgE/total IgE ratio and presence of comorbidities should be considered when performing OFC: for instance, a history of asthma or high values of allergen specific IgE are associated with a significantly higher risk of a severe reaction (37, 39–41).

Other factors might contribute to reaction severity, some of which have been termed augmentation or co-factors, such as exercise, infections, alcohol and medication (42).

In real life, the patient threshold level can be influenced by all these factors, as the case of gluten-induced anaphylaxis shows.

This clinical entity can be elicited during gluten challenge with high allergen doses, in the absence of cofactors, or with lower allergen doses in the presence of exercise and other augmentation cofactors, like acetylsalicylic acid or alcohol (43). In other words, augmentation factors might be necessary in order to achieve a reaction threshold, which would not otherwise be reached with a normal diet. From a nutritional standpoint, this means that the amount of gluten needed to elicit a reaction in real life may be much lower than the dose eliciting reaction during OFC, due to the presence of other factors, which are as yet only partially recognized.

A negative OFC test allows the tested food to be gradually reintroduced into the patient's diet at home.

However, even in the case of a positive OFC outcome, different practical indications may be provided to the child's family. If a reaction occurs after the ingestion of a very-low dose, the patient should be advised to follow a strict avoidance diet.

Conversely, if a clinical reaction occurs after the ingestion of a medium-high cumulative dose (which is allergen-specific), and all previous doses have been tolerated, the patient may be reasonably allowed to eat not only products containing traces of the allergen, but also foods that contain a lower amount of allergen than the one that elicited the symptoms.

Thereby, the role of the dietitian is crucial to calculate the exact amount of allergen ingested, and to advise parents which specific foods they can introduce into the child's diet.

For instance, in the case of a mild-moderate reaction occurring after the ingestion of hazelnuts (e.g., cumulative dose 5 g) during OFC, children and/or their parents may gradually introduce packaged products into the diet, such as biscuits or snacks, that contain hazelnut as an ingredient in a dose much lower than the dose eliciting symptoms during OFC (e.g., 1 or 2 g). For each packaged product, a careful reading of the ingredients on the label allows for quantifying the exact amount of hazelnut contained, and therefore to determine the portion that can be safely consumed by the child.

Even in the case of baked egg or milk, once the individual safe dose has been determined with OFC, the baked products of various brands may be compared with each other regarding the amount of egg and/or milk contained. Thus, the different quantities of “allowed” products are established, and the family may be advised to choose which to consume, alternating them.

With regard to dairy products, if during OFC the child tolerates the amount of 50 ml of regular milk as a cumulative dose, at home he will be advised to choose from the different dairy products with equivalent milk protein content (e.g., a teaspoon of aged cheese, 1/3 jar of yogurt, etc.).

### Dietary Habits and Preferences

To plan a personalized nutritional treatment for children with food allergies, it is also essential to investigate the eating habits of the children and his family.

The evaluation of dietary patterns provides more reliable data on real food intake than the assessment of single nutrients considered separately (44).

Actually, in recent years innovative food patterns are on the rise: vegetarian or vegan diets, consumption of exotic foods and “novel foods.”

The choice of a dietary style could have clinical implications, as it could lead to exposure to uncommon/atypical allergens.

For instance, lupine flour is frequently used in products intended for vegetarian and vegan nutrition, as it increases their protein content, or as a gluten substitute in baked goods.

Lupin is considered a major allergen in the EU and the UK, but not in the USA (5).

The consumption of exotic or typical oriental foods could increase exposure to seeds such as sesame, flaxseed, poppy seeds and mustard seeds, which are not considered as major allergens worldwide.

A careful investigation of eating habits is essential to understand if the food allergic child may be exposed to the risk of eating these foods (5).

Moreover, the child's food preferences should always be taken into account.

First, it should be considered that, in food allergic children, food habits develop in the context of their chronic pathology.

A higher risk of fussy eating and feeding difficulties has been reported in children who have followed an exclusion diet, compared to healthy children (45). In addition, it is not infrequent that, after a negative food challenge, food fear persists and the child remains reluctant to introduce the food that he has long been used to avoiding (46). For this reason, children and their parents should be encouraged to consume the tolerated foods after a negative challenge.

On the other side, a careful patient history should be taken before dietary interventions, as sensitization can be without clinical relevance.

### Nutritional Assessment and Patient—Tailored Nutritional Plan

The nutritional assessment of children with food allergies should include different steps, starting from the measurement of anthropometric parameters, followed by the estimation of the child's total energy expenditure [which is determined by resting energy expenditure (REE) and physical activity level], and an accurate collection of patient diet history.

The gold standard for the measurement of REE is indirect calorimetry (47).

Very few data exist on REE in food allergic children, since indirect calorimetry is time- consuming and requires specialized personnel (48). For this reason, in clinical practice REE is often estimated using predictive equations, easier to use but less specific (49).

To estimate the child's total energy expenditure (TEE) during the day, REE should be multiplied by a factor known as the Physical Activity Level (PAL).

Three levels of habitual physical activity are commonly considered, according to the child's lifestyle: light, moderate or vigorous (50).

The collection of a detailed diet history in allergic patients is the crucial step required to formalize patient-tailored advice (see Table 1).

**Table 1:** Focused diet-history in allergic patients.

• Single or multiple food allergy • Food amount provoking reactions • Symptoms every time food is eaten • Cultural and religious factors affecting dietary habits • Details and reasons for any avoided foods • Suspected allergen triggering reactions, other than those already known • Any current or previous treatment • Presence of any chronic condition that may further impair dietary intake • Any fortified foods or nutritional supplements being taken

To investigate the eating habits and the nutrient intake of the child, the number of daily meals and how they are distributed throughout the day should be recorded.

In addition, the child or his family should be asked which foods he has a preference or an aversion for and how a typical meal is composed.

Parents are asked to fill in a 3 or 7 day diary, recording all the foods and beverages consumed within the selected days, time of consumption, quantity consumed, and details about each food item (recipe and ingredients used, cooking method or brand name of a packaged product) (48).

Taking into account all this information, the dietitian is able to make a personalized nutritional plan which best fits the patient's needs.

Afterwards, food allergic children should be periodically re-evaluated, as an appropriate follow-up allows the practitioners to assess compliance to the diet and to ensure optimal growth (13). Following patients over time is also important to assess if natural tolerance to triggering food has been reached (4).

## Discussion

Important progress has been made in the understanding, diagnosing and treatment of food allergy in the last decade. The approach of nutritional management of food allergy has moved on from simply being “yes or no” to “how much?”, “in which form?” and “for which patients?”

OFC is changing from a reintroduction procedure to a more personalized one, aiming to identify the clinical reactivity of each patient. Thus, physician expectation is not simply to confirm or to exclude the diagnosis of food allergy, but also to determine which is the patient threshold dose and to establish if the child is able to tolerate baked products or low dose allergen.

In the age of precision medicine, tailored management of food allergy should be always targeted in clinical practice. To this purpose, the role of the dietitian is crucial to give patient tailored advice, considering patient threshold dose, dietary habits and preferences and baseline nutritional assessment.

In the near future it will be expected that other factors, like patient genetic information and microbiota signature could be taken into account in order to make a personalized nutritional plan.

## Author Contributions

ED: conceptualization. ED and EP: writing—original draft. GZ: review and editing. All the authors read and approved the final version of the manuscript.

### Conflict of Interest

The authors declare that the research was conducted in the absence of any commercial or financial relationships that could be construed as a potential conflict of interest.
